# Tribological and Mechanochemical Properties of Nanoparticle-Filled Polytetrafluoroethylene Composites under Different Loads

**DOI:** 10.3390/polym16070894

**Published:** 2024-03-25

**Authors:** Weixuan Lv, Tingmei Wang, Qihua Wang, Kian Kun Yap, Fuzhi Song, Chao Wang

**Affiliations:** 1 State Key Laboratory of Solid Lubrication, Lanzhou Institute of Chemical Physics, Chinese Academy of Sciences, Lanzhou 730000, China; lvweixuan21@mails.ucas.ac.cn (W.L.); wangqh@licp.cas.cn (Q.W.); 2 Center of Materials Science and Opto-Electronics Engineering, University of Chinese Academy of Sciences, Beijing 100049, China; 3 Tribology Group, Department of Mechanical Engineering, Imperial College London, London SW7 2BX, UK; kkyap@imperial.ac.uk; 4 Qingdao Center of Resource Chemistry & New Materials, Qingdao 266100, China

**Keywords:** polytetrafluoroethylene, nanoparticles, load, tribofilm

## Abstract

For the tribological properties of nanoparticle-modified PTFE, a more comprehensive study has been conducted, but there is still some room for research on tribology behavior, tribofilm formation and structure evolution of polytetrafluoroethylene (PTFE) filled with α-Al_2_O_3_ and SiO_2_ nanoparticles during sliding against steel counterparts under different loads. At the same time, it establishes the linkage and mechanism between the maintenance of mechanical strength and the tribological application of polymers in service and provides corresponding scientific data and theoretical guidance for the long-lasting application of polymer lubrication materials. It is found that both composites exhibit good wear resistance across the pressure of 1 MPa to 10 MPa, with the α-Al_2_O_3_/PTFE composite demonstrating better performance stability compared to the SiO_2_/PTFE composite. The high wear resistance is attributed to the formation of tribofilms at the friction interface. For the α-Al_2_O_3_/PTFE, an island-like tribofilm is formed with a thickness ranging from 100 to 200 nm, while the tribofilm of the SiO_2_/PTFE composite is thinner, measuring approximately 50 to 100 nm, and manifests a striped pattern. The chemical composition, both at the surface and subsurface levels, as well as the morphology of the tribofilms, were studied using FTIR spectrometry, X-ray photoelectron spectroscopy (XPS), and FIB-TEM. It is found that the difference in thickness and microstructure of the tribofilms for the two composites is mainly due to the tribochemistry of the nanoparticles. The α-Al_2_O_3_ nanoparticle plays a “cohesion” role during the formation of the tribofilm, which facilitates the formation of a thicker, more uniform, and stronger adhered tribofilm on the metallic counterpart, making it more robust against higher shear stress.

## 1. Introduction

Solid lubrication technology, serving as a complementary approach to fluid lubrication, primarily utilizes solid lubricants to lubricate friction interfaces. It offers a broader temperature range applicability and is well suited for demanding environments such as vacuum, high temperatures, and dry friction [1,2]. Among the polymers that serve as excellent solid lubricating materials, polytetrafluoroethylene (PTFE) stands out as a self-lubricating material with good chemical stability [3]. However, its high wear rate (10^−3^~10^−4^ mm^3^ (Nm)^−1^) makes it impossible to use it in a pure polymer form for practical applications, and it is necessary to enhance its friction-reducing and anti-wear capabilities by adding various fillers [4]. Tanaka et al. [5] conducted a study on the wear resistance of PTFE filled with different fillers such as glass fiber (GF), bronze, ZrO_2_, TiO_2_, MoS_2_, graphite, etc., which greatly improved the wear resistance of PTFE. Zhang et al. [6] compared the friction and wear properties of PTFE composites in different lubricating mediums and concluded that the presence of the lubricating mediums greatly influences the tribological properties of the composites.

From the perspective of solid mechanics, the improvement of wear resistance of polymer materials by fillers mainly comes from the improvement of mechanical properties [7]. Blanchet et al. [8,9] proposed the load support theory by means of mechanical modeling and suggested that wear-reducing fillers are the main wear-reducing mechanism to resist the expansion of surface cracks. Even though nano-fillers are not as effective as micrometer fillers to inhibit surface cracks on materials. In recent years, studies still show that nano-fillers (Aluminium oxide, silicon oxide, graphene, etc.) can still reduce the wear rate of PTFE by 3–4 orders of magnitude [10,11]. Krick et al. [12] and Bhargava et al. [13] found that nanofillers not only enhanced the mechanical properties of PTFE but also facilitated tribochemical reactions. These reactions resulted in the formation of carboxylated tribofilms at the friction interface. Moreover, the formation of these tribofilms reduced direct contact between the polymer material and the counterpart, consequently decreasing the adhesive wear of softer materials [14,15].

Under dry friction conditions, many polymer materials form tribofilms, which have been shown to be transferred from the material to the interface of the counterpair, forming an adhesive coating that alters the contact state [16,17]. Typically, the tribofilm shifts the counterpart from metal–polymer sliding to polymer–polymer sliding, thereby enhancing friction reduction and wear resistance. Xie et al. [18] measured the thickness of the tribofilm generated on the steel surface through the friction and wear experiments of Ni/PTFE composites sliding against steel and found that the tribofilm thickness increased with the sliding speed, and increased first and then decreased with increasing load. In addition, along with the increase in the tribofilm thickness, the coefficient decreased and the wear volume increased. Using nanomechanical tests, Onodera [19] and Ye et al. [20,21,22] found that the presence of polar interactions at the frictional interface of PTFE composites facilitates the adsorption of the tribofilm and the enhancement of internal interactions. Meanwhile, the strong adsorption of tribofilms on the interface of counterpair is associated with the tribochemistry at the interface [23]. Harris et al. [24] suggested that the tribofilm adhesion and load-carrying capacity resulting from these tribochemical reactions were effectively improved, thus reducing the wear of composites during sliding against steel counterparts.

At present, there are many reports on the tribological properties of nanoparticle-filled PTFE composites, while the kinetic process and mechanism of tribofilm formation are less explored, especially the tribochemical reaction process influenced by load and nanoparticles as well as the intrinsic relationship among properties of nanofiller, the microstructure of tribofilm and the tribological behaviors, which still lack an in-depth understanding. In this paper, the tribology behavior, tribofilm formation and structure evolution of PTFE filled with α-Al_2_O_3_ and SiO_2_ nanoparticles during sliding against steel counterparts under different loads were studied. It is the aim of this work to reveal the impact of nanoparticles’ properties on the tribological behaviors and tribofilm property structures of the hybrid PTFE composites under different normal loads. It is also the goal of this work to gain deeper insight into the tribological mechanisms and tribofilm formation of the nanoparticles.

## 2. Experimental

### 2.1. Material Preparation

Aluminum oxide (α-Al_2_O_3_, ≥99%, particle size 30 nm) was provided by Shanghai Macklin Biochemical Technology Co., Ltd. (Shanghai, China), has strong heat resistance, good formability, stable crystal phase, high hardness, good dimensional stability, and can be widely used in a variety of plastics, rubber, ceramics, refractory materials, etc., to strengthen and toughen the products. Silicon oxide (SiO_2_, ≥99%, particle size 15 nm) was provided by Shanghai Macklin Biochemical Technology Co., Ltd. (Shanghai, China), is chemically stable, does not react with water, and has high fire resistance, high temperature resistance, low coefficient of thermal expansion, high insulation, corrosion resistance, piezoelectric effect, resonance effect, and its unique optical properties. Polytetrafluoroethylene (PTFE, 100%) was provided by Daikin Fluorochemicals Co., Ltd. (Changshu,China), has excellent chemical stability, corrosion resistance, sealing, high lubrication and non-stick, electrical insulation and good anti-aging resistance. GCr15 bearing steel was provided by Changzhou Yapai Automation Bearing Enterprise Store(Changzhou,China),has high hardness and wear resistance high dimensional stability and small heat treatment deformation.

Polymer sample preparation: aluminum oxide and PTFE were weighed in a 5:95 mass ratio and then mixed in a high-speed mixer. After mixing for 30 s each time, the mixer was placed in a freezer to cool for 10 min. This process was repeated 5 times. The mixed powder mixture was compressed in an 80 × 50 mm rectangular mold to approximately 50 MPa in a hydraulic press. The sample was sintered in an oven. Finally, the sintered samples were cut into 4 mm × 4 mm × 10 mm rectangular cylindrical samples for subsequent tests. Similarly, the preparation of PTFE composites with 5wt% silicon oxide nanoparticles was obtained. The density of the α-Al_2_O_3_/PTFE/sample was measured to be 2.26 g/cm^3^, while the density of the PTFE/SiO_2_ sample was found to be 2.21 g/cm^3^.

Metal counterpart preparation: The counterpart used in this experiment was a flat, circular (∅25 mm × ∅25 mm × 3 mm) plate of GCr15 steel. Firstly, the counterpart was put into petroleum ether for ultrasonic cleaning and then pre-polished with 800 mesh and 2000 mesh sandpaper, respectively, and finally, the diamond abrasive polishing paste with grain size of w0.5 was used to perform mirror polishing on a polishing cloth. The roughness of the samples was controlled to be approximately 0.01 μm. The polished counterpart was ultrasonically cleaned in petroleum ether, wiped with non-woven cloth and stored in a vacuum drying box.

### 2.2. Tribological Tests

**Figure 1:**
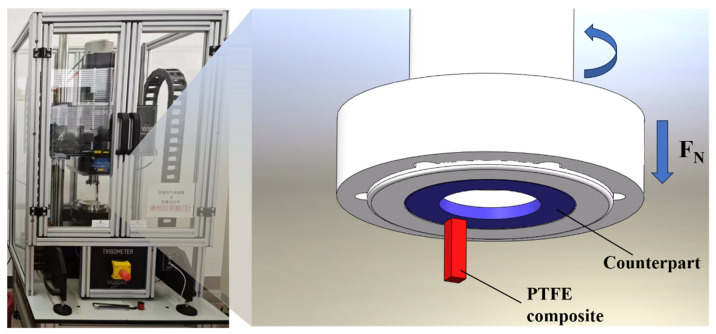
Schematic diagram of a pin-on-disc friction and wear testing machine.

The friction test was performed on the tribometer in a pin-on-disc configuration under different loads, as shown in Figure 1. The upper specimen is a GCr15 steel counterpart and the lower specimen is a PTFE composite of 4 mm × 4 mm × 10 mm rectangular cylindrical sample. The test environment maintained a temperature of 25 °C and a humidity level of 30%. The detailed friction test conditions were as follows: a sliding speed of 120.95 mm/s (equivalent to a rotational speed of 70 rpm with a rotational radius of 16.5 mm), and a duration time of 180 min. Friction tests were conducted under seven different loads of 1, 2, 3, 4, 6, 8, and 10 MPa. To ensure accuracy, each group of samples underwent at least three parallel repetitions of the tests to calculate the average friction coefficient and wear rate. Before and after the friction test, the PTFE samples were weighed five times using an analytical balance (Precisa XB220A, Dietikon, Switzerland), and the mass loss $m_{loss}$ was calculated by taking the average value (accuracy ± 10 μg). The wear rate was calculated according to Equation (1).

(1) $$k=\frac{m_{loss}\;}{\left(\rho \times F_{N}\times s\right)}$$

where $F_{N}$ is the normal force; ρ is the density of the test sample; s is the sliding distance, which is calculated by Equation (2).

(2) $$s=r_{min}\times \pi \times d\times t$$

where $r_{min}$ is the rotational speed per minute, d is the radius of rotation, and t is the friction time.

### 2.3. Characterization of Tribofilm

At the end of the friction wear test, the steel ring was cut into samples with a length and width of no more than 20 mm. The morphology of the tribofilm on the surface of the steel counterpart and the wear scar of the polymer samples was observed using a scanning electron microscope (SEM, JSM-7610F, Akishima-shi, Japan). A 3D image of the tribofilm was obtained using a confocal microscope (CLSM, VT6000, Zhongtu Instrument, Shenzhen, China). For each sample, three profile lines were drawn, and the cross-sectional height of 3 segments of the tribofilm along each profile line was measured. Subsequently, the average values were calculated. Finally, based on these data, the average thickness of the tribofilm on the surface of the steel counterpart under different loads was determined.

In order to investigate the mechanism of the evolution of the tribofilm formation, attenuated total reflection Fourier infrared spectroscopy (ATR-FTIR, TENSOR 27, Billerica, MA, USA) and X-ray photoelectron spectroscopy (XPS, Thermo Fisher Scientific, Waltham, MA, USA) were used to detect the chemical composition of the tribofilm. Meanwhile, in order to further understand the formation process, the internal structure of the tribofilm was characterized using a Transmission Electron Microscope (TEM) combined with Selected Area Electron Diffraction (SAED) or Scanning Transmission Electron Microscopy attached Energy Dispersive X-ray Spectroscopy (STEM/EDS) (Tecnai G2 TF20 S-TWIN, FEI Waltham, MA, USA). The TEM specimens of the tribofilms formed on the steel counterfaces were prepared by a Focused Ion Beam (FIB) equipped on a DualBeam SEM-FIB instrument (FEI Quanta 3D, Potsdam, German). Prior to acquiring cross-sectional slices, the area of interest was coated with a platinum cap layer deposited on FIB by ion-beam-assisted deposition to form a gaseous organic Pt compound.

## 3. Results and Discussion

### 3.1. Friction Test Results

**Figure 2:**
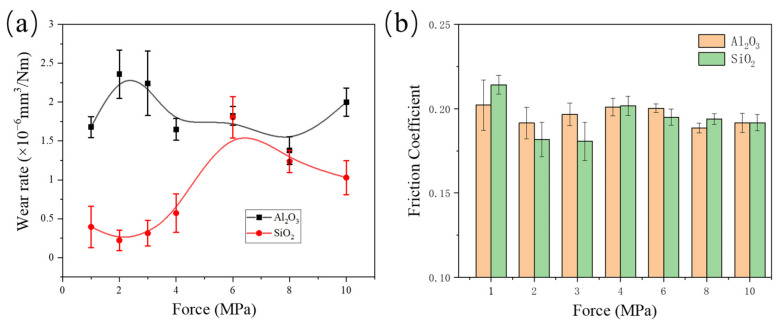
Wear rate and friction coefficient of different PTFE composites at different loads: (**a**) Wear rate, (**b**) Friction coefficient.

Figure 2a illustrates the trend of wear rates for 5wt% α-Al_2_O_3_/PTFE and 5wt% SiO_2_/PTFE under different loads. While there is a slight variation in the wear rate of α-Al_2_O_3_/PTFE with increasing load from 1 MPa to 10 MPa, the overall wear rate remains within the same order of magnitude, approximately 10^−6^ mm^3^/Nm. This suggests that the change in load does not significantly impact the wear resistance of α-Al_2_O_3_/PTFE. In contrast, although the wear rate of SiO_2_/PTFE composite is lower than that of α-Al_2_O_3_/PTFE in the range of tested loads from 1 to 10 MPa, it notably increases with the rise in load, from 2.5 × 10^−7^ mm^3^/Nm to 3 × 10^−6^ mm^3^/Nm. This result suggests that the α-Al_2_O_3_/PTFE composite has a better wear-resistance performance stability compared to SiO_2_/PTFE across a wide range of loading conditions.

From Figure 2b, it can be observed that although different nanoparticles have varying effects on the wear rate of PTFE composite materials, their influence on the variation in friction coefficients is minor. This is related to the fact that the friction coefficient of PTFE composites depends on the orientation of the polymer molecular chains at the friction interface [25]. Therefore, there is little difference in the friction coefficients of the two PTFE composite materials, both being around 0.2. Additionally, it can be observed that the error bars of the friction coefficients of both PTFE composite materials under high loads are smaller than those under low loads, indicating the contact between the materials and counterpart tends to stabilize with the increasing load.

### 3.2. Tribofilm Morphology

**Figure 3:**
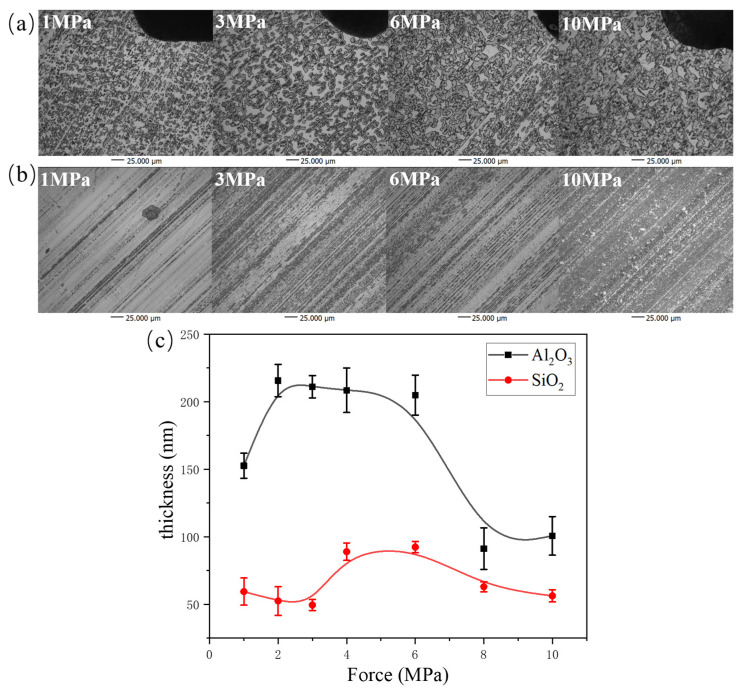
Confocal surface morphology and thickness variation of different PTFE composite tribofilms formed on the metal counterparts under different loads: (**a**) α-Al_2_O_3_/PTFE, (**b**) SiO_2_/PTFE, (**c**) thickness variation.

In order to investigate the surface morphology and thickness of the tribofilm formed on the surface of the counterpart, the image acquisition was carried out by confocal microscopy. Figure 3a shows the morphology of the tribofilm formed by α-Al_2_O_3_/PTFE rubbing against steel. It can be clearly seen that with the increase in load, the morphology of the transfer film changes from fine particles to increased coverage and mutual connection, gradually transitioning into irregular island shapes. For the SiO_2_/PTFE composite, as shown in Figure 3b, the morphology of its transfer film appears as elongated stripes under low loads and gradually expands to both sides with increasing load, eventually forming a complete transfer film at higher loads.

The height of the tribofilm was measured by confocal 3D scanning, and the average value was taken to calculate the thickness of the tribofilm. As shown in Figure 3c, the thickness of the transfer film formed by α-Al_2_O_3_/PTFE varies approximately between 150 nm and 250 nm. Its thickness increases initially with the increase in load, then keeps constant, and finally decreases. This trend indicates that the transfer film is relatively stable within the load range of 2–6 MPa. On the other hand, the thickness of the transfer film formed by SiO_2_/PTFE ranges from 40 nm to 100 nm, which is smaller than that of α-Al_2_O_3_/PTFE. With the increase in load, the thickness initially increases and then decreases. The thickness is at its maximum at 6 MPa, at which point the material exhibits the highest wear rate (Figure 2a).

**Figure 4:**
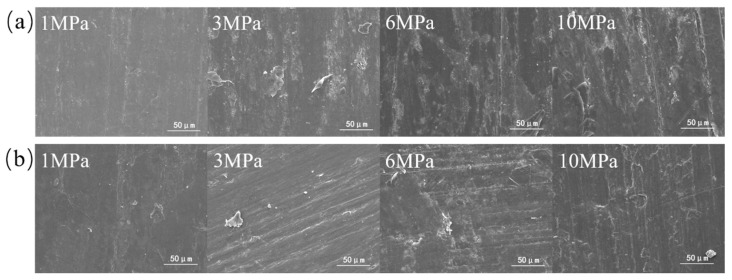
Scanning electron micrographs of the worn surfaces of the PTFE composites under different loads: (**a**) α-Al_2_O_3_/PTFE, (**b**) SiO_2_/PTFE.

Figure 4 shows the wear morphology of α-Al_2_O_3_/PTFE and SiO_2_/PTFE composite materials after rubbing against steel under different loads. It can be observed that under low loads, the surface of the α-Al_2_O_3_/PTFE composite material exhibits a small number of scratches and debris. As the load increases, the debris is compacted, accumulated, and adhered to the material surface. In contrast, for the SiO_2_/PTFE composite material shown in Figure 4a, at 1 MPa, the surface appears relatively smooth. However, with the increase in load, noticeable scratches appear on the surface, indicating abrasive wear mechanisms.

### 3.3. Analysis of Tribochemical Reactions

**Figure 5:**
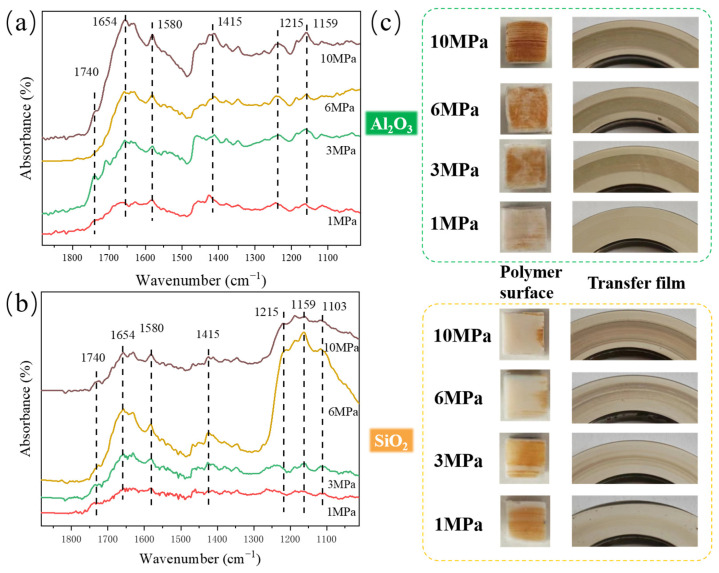
ATR-FTIR spectra of the tribofilms formed on the metal counterparts and the corresponding worn surface images: (**a**) α-Al_2_O_3_/PTFE, (**b**) SiO_2_/PTFE, (**c**) worn surface images.

To investigate the chemical composition changes in transfer films on the steel counterparts with increasing load, FTIR spectroscopy analysis was conducted. As shown in Figure 5a, in the transfer film formed on the steel surface during the sliding of α-Al_2_O_3_/PTFE composite, peaks at 1159 cm^−1^ and 1215 cm^−1^ corresponding to the -CF_2_- in PTFE are observed. Additionally, peaks related to carboxylate salts generated from tribochemical reactions are also evident at 1415 cm^−1^ and 1654 cm^−1^ [26,27]. Furthermore, it can be shown the ratio of the intensity of the carboxylate peak to the C-F peak is positively correlated with the load, indicating that as the load increases, the degree of tribochemical reactions intensifies. This result corresponds to the photographs of worn surfaces of the α-Al_2_O_3_/PTFE composite and steel counterpart shown in Figure 5c.

Figure 5b shows the FTIR spectrum of the transfer film formed on the counterpart surface after sliding against the SiO_2_/PTFE composite. Similar to α-Al_2_O_3_/PTFE, peaks related to the -CF_2_- in PTFE and peaks associated with tribochemical reactions with carboxylate end groups are also observed [28]. However, a notable difference is observed when the load increases to 6 MPa; the intensity of the -CF_2_- in PTFE significantly increases, and a peak at 1103 cm^−1^ attributed to Si-O appears on the right side of the peak, indicating the presence of a significant amount of PTFE and SiO_2_ transfer on the counterpart surface. From the photographs of the worn surfaces of SiO_2_/PTFE samples in Figure 5c, it can be seen that under lower loads, the SiO_2_/PTFE surface adheres to obvious brown tribochemical products, which essentially disappear under the frictional shear action of higher loads. This result is consistent with the FTIR result.

In addition, comparing the intensity of the carbonyl peak of the tribofilms formed by the two PTFE composites, it can be inferred that the tribochemical reaction between α-Al_2_O_3_/PTFE and the metal counterpart is more active. This leads to the generation of more polar functional groups in the transfer film, enhancing the adhesion between the carboxyl end groups and the metal counterpart, thereby forming a thicker and denser transfer film [24]. On the other hand, the thickness of the transfer film of SiO_2_/PTFE reaches its maximum as the load increases, and then PTFE transfer becomes predominant. This may also be the main reason for the increase in the wear rate of SiO_2_/PTFE under high loads.

**Figure 6:**
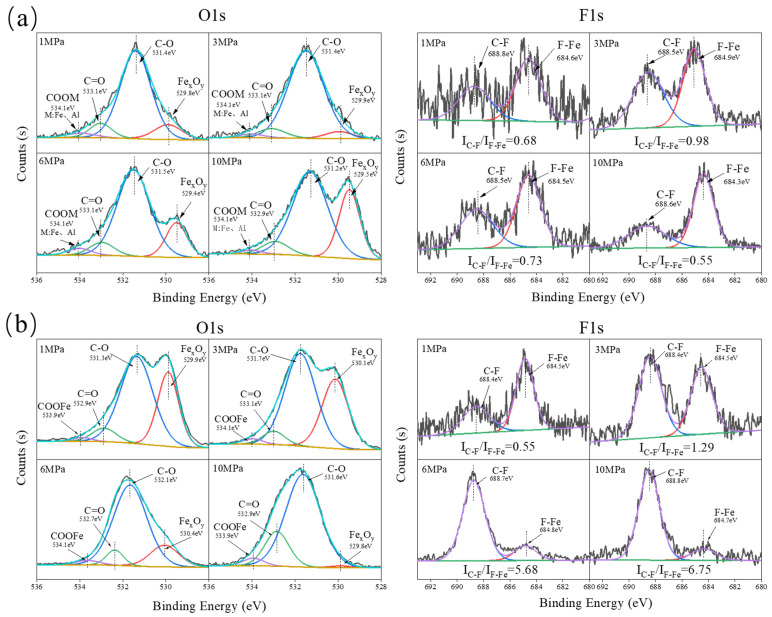
XPS spectra of tribofilms formed on the metal counterparts under different loads: (**a**) α-Al_2_O_3_/PTFE, (**b**) SiO_2_/PTFE.

Figure 6a displays the O1s and F1s spectra of the transfer film formed on the metal surface after sliding against α-Al_2_O_3_/PTFE composites under different loads. From the O1s spectrum, peaks corresponding to C-O and C=O bonds can be observed, indicating significant tribochemical reactions occurring in PTFE [29]. This result is consistent with the FTIR spectrum results and with previous literature reports. In previous studies, PTFE reacted with water and oxygen in the air to produce functional groups such as COOH, which then chelate to the surface of the steel counterpart and α-Al_2_O_3_ nanoparticles [30,31]. We attempted to identify characteristic peaks of corresponding chelated salts in the O1s spectrum, but due to overlap with the C-O peak, it was difficult to clearly distinguish their respective positions. Nevertheless, a distinct peak at 684 eV in the F1s spectrum suggests the formation of F-Metal bonds (F-Fe or F-Al), while significant iron oxide can also be observed in the O1s spectrum [24]. Both peaks show an increasing trend in intensity with increasing load, indicating a positive correlation between tribochemical reactions and load.

Figure 6b shows the spectra of O1s and F1s of the tribofilms formed on the steel counterpart after sliding against SiO_2_/PTFE composites under different loads, which show great similarity to that of α-Al_2_O_3_/PTFE tribofilms. However, there is an opposite trend in the intensity of peaks such as Fe_x_O_y_ and F-Fe with increasing load. Specifically, as the load increases, the extent of tribochemical reactions gradually decreases. This is particularly evident from the variation in the intensity ratio of F-Fe to C-F bonds. Under low loads, the composition of the transfer film is mainly dominated by tribochemical reactions such as chelated salts, F-Metal and Fe_x_O_y_, while under high loads, the composition of the transfer film is mainly attributed to the transfer of PTFE. This result is consistent with the infrared spectrum results.

Additionally, comparing the XPS spectra of the tribofilms for the two PTFE composites, it can be seen that SiO_2_ contributes more to the formation of tribofilms with a high degree of tribochemical reactions at low loads compared to α-Al_2_O_3_. This could be attributed to the higher hardness of α-Al_2_O_3_ particles, which may cause greater abrasion damage to the tribofilm. However, under high load, the strength and interface bonding of the tribofilm formed by SiO_2_ are insufficient to resist the shear stress. When the load exceeds 6 MPa, the tribofilm becomes thinner and may even be destroyed, and only the cyclic transfer and removal of PTFE friction transfer film occur, leading to an increase in wear rate. In contrast, the participation of α-Al_2_O_3_ in the tribochemical reaction strengthens the stability of the tribofilm, which is able to withstand the shear under a higher load, and the wear rate is maintained at a lower level. Nevertheless, the thickness of the tribofilm still shows a tendency to decrease due to the high shears the load is higher than 6 MPa.

**Figure 7:**
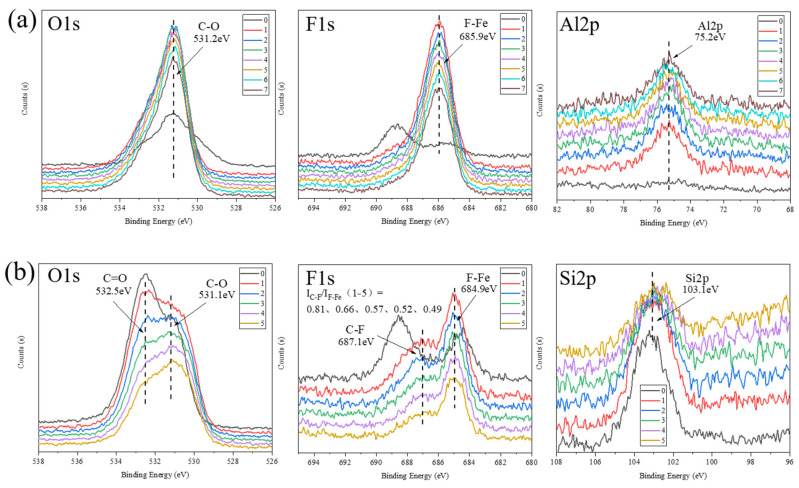
XPS depth profiles of tribofilms formed on the metal counterparts under 6 MPa load: (**a**) α-Al_2_O_3_/PTFE, (**b**) SiO_2_/PTFE.

To further understand the chemical composition changes within the transfer films for the α-Al_2_O_3_/PTFE and SiO_2_/PTFE composites, the topmost XPS spectrum was collected on the tribofilms formed under 6 MPa pressure. Subsequently, sequential Ar+ ion etching was performed at the same location for seven cycles and five cycles, with each etching cycle having a depth of 29 nm. XPS spectrum was collected simultaneously during each etching cycle. As shown in Figure 7 of the set of spectra, the higher the spectrum’s number, the bigger the depth from which it was recorded.

In the O1s spectra, compared to the surface, the C-O bond intensity of the tribofilms of the two composites shows a significant increase. Particularly for α-Al_2_O_3_/PTFE, the spectrum shape undergoes a complete change, with a weakening of the C=O and Fe-O peaks, presenting a stronger single peak of C-O. While the shape of the O1s spectrum of SiO_2_/PTFE transfer film remains unchanged, the intensity ratio of C-O to C=O increases with the increase in etching depth. In the F1s spectra, it can be seen that there is a large amount of C-F on the surface, but the intensity ratio of C-F to F-Fe in the tribofilm of SiO_2_/PTFE composite decreases continuously from 0.81 to 0.49 as the etching depth increases. The tribofilm of α-Al_2_O_3_/PTFE, on the other hand, shows a more obvious difference after removing the surface components, with the C-F peak almost disappearing and showing a strong single peak of F-Metal (F-Fe or F-Al), indicating the internal tribofilms is mainly composed of uniform tribochemical reaction products. It should be noted that the F-Metal peak was attributed to the mixture of F-Fe and F-Al mainly because, compared with the pre-etching, the peak position has changed from 684.9 to 685.9, which does not exactly correspond to the pure F-Fe bond (see the F-Fe peak in Figure 7b). In the Al2p spectra, the alumina nanoparticles are uniformly distributed inside the tribofilm and less appear on the surface, indicating that they are involved in the formation of the tribofilm; whereas the silica nanoparticles are more distributed on the surface of the tribofilm, which will be taken away during sliding along with the abrasive debris at any time, and their participation in the tribochemical reaction is small, and most of them are still acting as abrasive particles on the friction interface.

**Figure 8:**
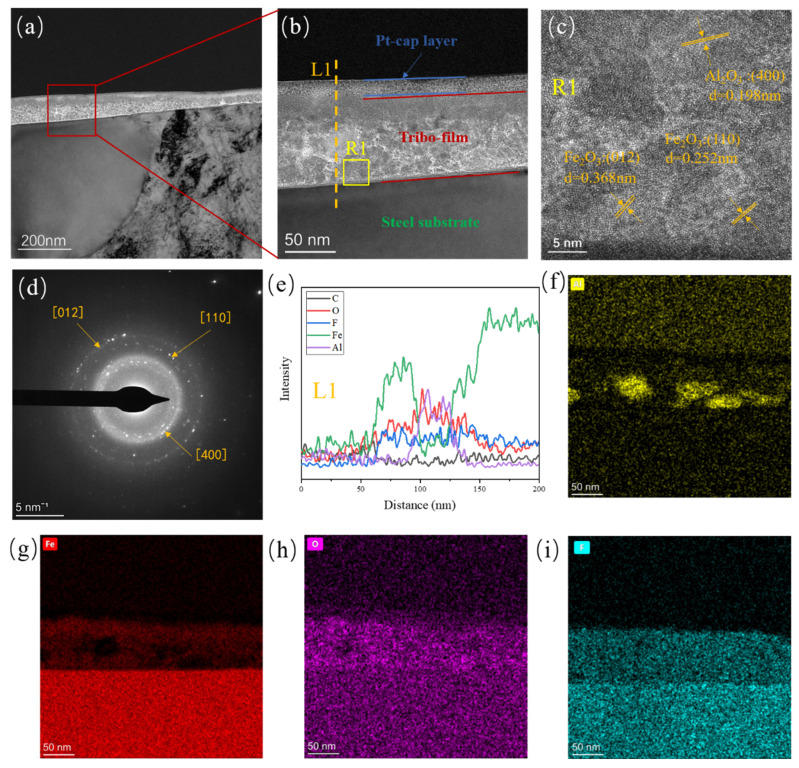
FIB high-resolution TEM images, diffractograms and EDS elemental analysis of the α-Al_2_O_3_/PTFE tribofilm formed on the metal counterpart under 6 MPa load: (**a**–**c**) high-resolution electron microscopy images, (**d**) diffractograms in R1, (**e**) EDS elemental intensity of L1, (**f**–**i**) EDS elemental intensity in (**b**).

FIB-TEM was used to further investigate the structure and chemical composition distribution of the tribofilms. Figure 8a–c show the morphology of the tribofilm on the surface of its metallic counterpart after sliding against α-Al_2_O_3_/PTFE composite. It can be seen that the tribofilms with a thickness of about 100 nm formed between the platinum coating and the steel counterpart. The tribofilm is divided into two layers; the upper layer is composed of mainly iron oxide, and the lower layer consists of a mixture of iron oxide, alumina particles, and carbon oxides. The C, O, F, Fe, and Al elements in the tribofilm can be seen from the EDS line sweep of Figure 8e. From the high-resolution image in Figure 8c and the diffracted spot in Figure 8d, the lattice stripe spacing and lattice parameter measurements reveal that the crystals with a lattice spacing of 0.198 nm in Figure 8c correspond to alumina, as indicated by the white region in Figure 8b. Additionally, the structures with lattice spacings of 0.252 nm and 0.368 nm are identified as iron oxide [29,32], corresponding to the grey region in Figure 8b. Meanwhile, combined with the EDS surface scan images of Figure 8f–i, it can be observed that alumina nanoparticles, as well as Fe, O, and F elements, are uniformly distributed within the tribofilm.

**Figure 9:**
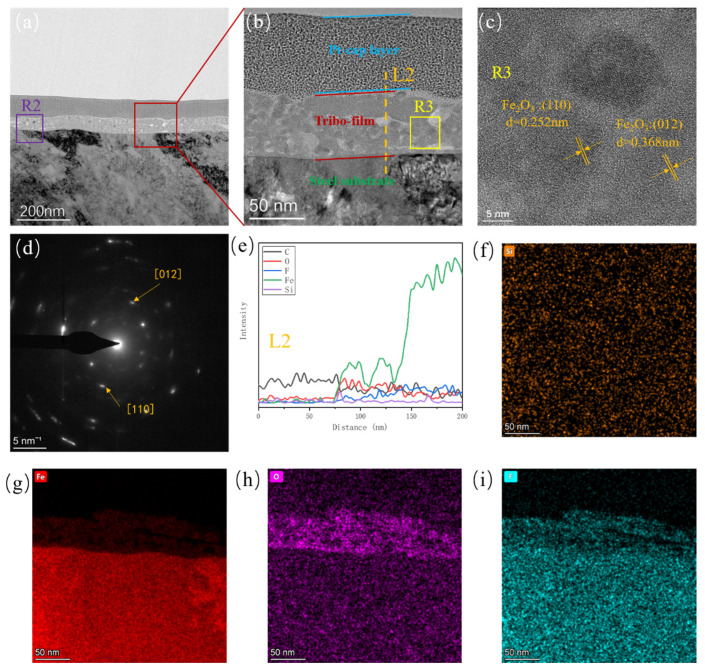
FIB high-resolution TEM images, diffractograms and EDS elemental analysis of the SiO_2_/PTFE tribofilm formed on the metal counterpart under 6 MPa load: (**a**–**c**) high-resolution electron microscopy images, (**d**) diffractograms in R3, (**e**) EDS elemental intensity of L2, (**f**–**i**) EDS elemental intensity in R2.

Figure 9a,b shows the FIB TEM of the tribofilm on the surface of the metallic counterpart after sliding against the SiO_2_/PTFE composite. The thickness of the tribofilm is approximately 60 nm, which is thinner and less dense compared to that of α-Al_2_O_3_/PTFE, consistent with the confocal measurement results. In Figure 9c, crystals with lattice spacings of 0.252 nm and 0.368 nm are identified as Fe_2_O_3_. From the EDS line and surface scan images of Figure 9e–i, it is evident that the tribofilm contains elements C, O, F, Fe and Si. However, it should be noted that the silicon element content is relatively low, and no significant silica nanoparticles were observed. This might be due to silicon oxide not participating in tribochemistry like alumina, existing randomly in the tribofilm, or being extruded from the friction interface as debris under shear stress.

### 3.4. Tribofilm Formation Mechanism

**Figure 10:**
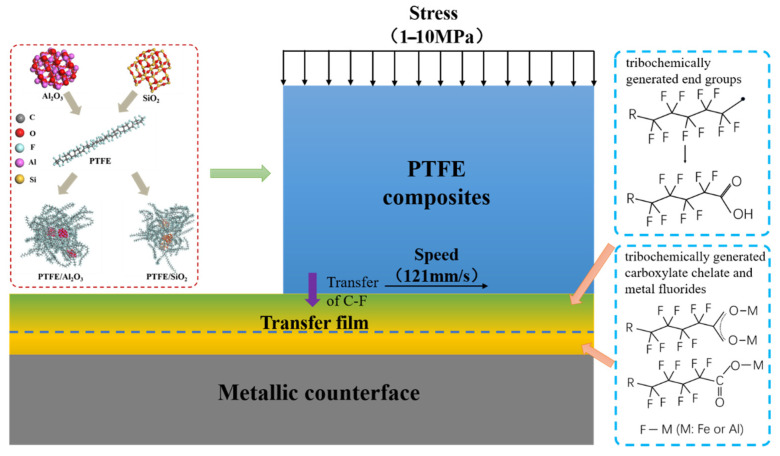
Schematic diagram of the tribofilm formation mechanism.

Based on the above results, a possible mechanism of mechanochemical reactions and tribofilm formation between PTFE composite materials and metallic counterparts is addressed, as shown in Figure 10. Combined with previous reports, this study further verifies the tribochemical reactions of nanoparticle-filled PTFE composites during sliding against metallic counterparts and elucidates the internal structure and dynamic formation process of the tribofilm. The main tribochemical reaction products include PTFE carboxylic acid, chelates between carboxylic acid end group and metals, metal fluorides generated from the reaction between small molecule fluorides and metal oxides, as well as metal oxides, etc. We can also find similar reaction products in Onodera’s findings [23]. These reaction products effectively promote the formation of the tribofilm. For α-Al_2_O_3_/PTFE, the transfer film exhibits a dual-layer structure, where the upper layer comprises a mixture of PTFE transfer and products of tribochemical reaction, while the lower layer mainly consists of uniform products of tribochemical reaction without obvious PTFE transfer. In contrast, for SiO_2_/PTFE, the transfer film presents a gradient structure overall. As the depth increases, there is progressively less PTFE transfer, while the amount of tribochemical reaction products gradually increases.

The formation of the tribofilm is related to the involvement of nanofillers in tribochemistry. This is consistent with Krick et al. [12] and Bhargava et al.’s [13] findings of promoting friction chemistry when nanofillers modify PTFE. For the α-Al_2_O_3_/PTFE composite, both Fe in the metallic counterpart and α-Al_2_O_3_ in the composite participate in tribochemistry. Particularly, α-Al_2_O_3_ plays a “cohesion” role in achieving reinforcement within the tribofilm. On the one hand, it facilitates thicker growth of the tribofilm, and on the other hand, it can disperse across the depth of the tribofilm, making it more robust against higher shear stress. In contrast, the tribochemical reactions for SiO_2_/PTFE composite are limited to the surface of the metal counterpart. The chemical composition of the tribofilm varies gradiently with changes in the thickness. Therefore, its resistance to shear stress is relatively weak, quickly reaching an equilibrium state of wear and regeneration. Consequently, the resulting tribofilm tends to have a thinner thickness.

## 4. Conclusions

In this paper, the tribology behavior, tribofilm formation and structure evolution of α-Al_2_O_3_ and SiO_2_ nanoparticle-filled PTFE composites under different loads were investigated, and the main conclusions are as follows:

1. Both composites exhibit good wear resistance under the pressure ranging from 1 MPa to 10 MPa. The wear rate of the SiO_2_/PTFE composite is generally lower than that of the α-Al_2_O_3_/PTFE; however, the α-Al_2_O_3_/PTFE composite demonstrates better performance stability compared to the SiO_2_/PTFE. For the α-Al_2_O_3_/PTFE, an island-like tribofilm is formed with a thickness of 100 to 200 nm, while the tribofilm of SiO_2_/PTFE composite is thinner, approximately 50 to 100 nm, and displays a striped pattern.
2. For the α-Al_2_O_3_/PTFE composite, the degree of tribochemical reactions shows a positive correlation with the load. Conversely, in the case of the SiO_2_/PTFE composite, the degree of tribochemical reactions gradually decreases as the load increases. For α-Al_2_O_3_/PTFE, the transfer film exhibits a dual-layer structure across depth, where the upper layer comprises a mixture of PTFE transfer and products of tribochemical reaction, while the lower layer mainly consists of uniform products of tribochemical reaction without obvious PTFE transfer. In contrast, for SiO_2_/PTFE, the transfer film presents a gradient structure. As the depth increases, there is progressively less PTFE transfer, while the amount of tribochemical reaction products gradually increases.
3. The difference in thickness and microstructure evolution of the tribofilms for the two composites is mainly attributed to the tribochemistry of the nanoparticles. The α-Al_2_O_3_ nanoparticle plays a “cohesion” role during the formation of the tribofilm. which facilitates the formation of a thicker, more uniform, and stronger adhered tribofilm on the metal counterpart, making it more robust against shear stress. This provides guidance for the study of tribological properties of nanofiller-modified PTFE under different operating conditions.

## Data Availability

The data that support the findings of this study are available upon request from the corresponding author. The data are not publicly available due to privacy and legal.
